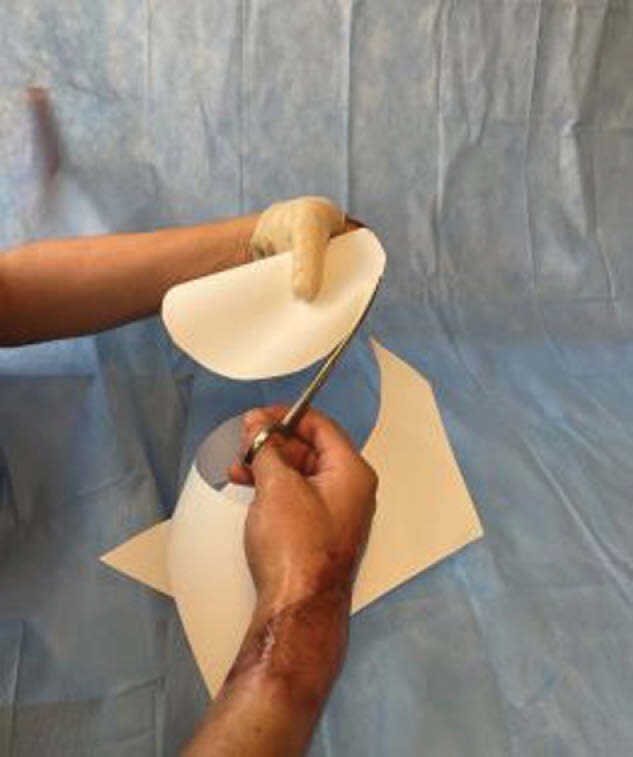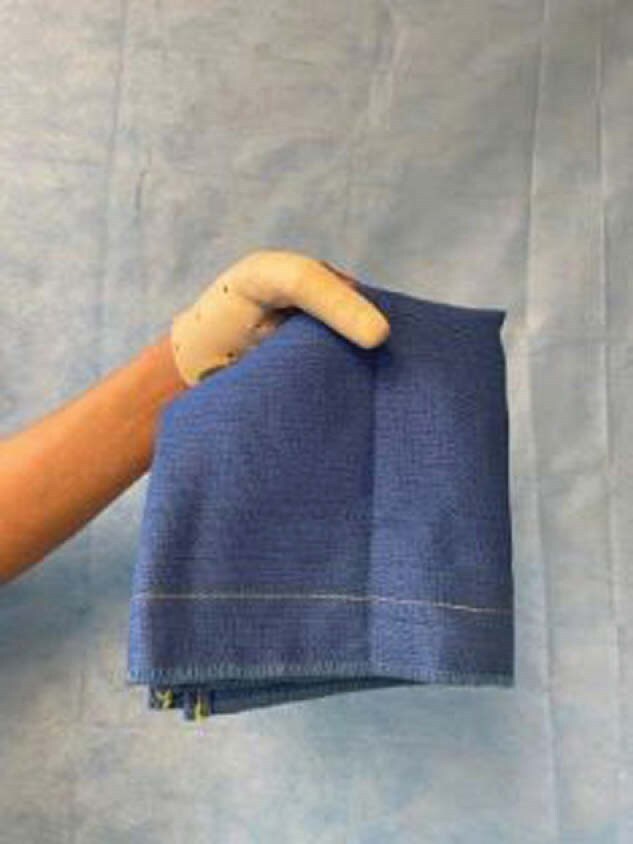# 997 Case Study: Opposition Splint for Thumb Amputation and Its Effectiveness in Decreasing Disability

**DOI:** 10.1093/jbcr/iraf019.528

**Published:** 2025-04-01

**Authors:** Sandra Montelongo, William Scott Dewey

**Affiliations:** United States Army Institute of Surgical Research Burn Center; United States Army Institute of Surgical Research Burn Center

## Abstract

**Introduction:**

Patient is a 27-year-old male with 6.6% TBSA burns sustained by contact with an electrical wire of unknown voltage. His surgical procedures included grafting and amputations of his digits (left thumb and index finger) and toes. He is currently unemployed and has limited funding and transportation. Due to lack of resources and his report of requiring assistance with daily activities, an opposition splint was fabricated to increase his manual function and independence. To determine the effectiveness of the splint, the patient completed an upper extremity outcome measure before and after a period of use.

**Methods:**

During his initial therapy session, the patient used his intact right dominant hand to complete the Spanish version of the QuickDASH Outcome Measure. This assessment is widely used to measure disability in patients who have had an injury to their upper extremity. A custom thermoplastic opposition splint was fabricated, an intervention previously shown to be effective as an interim option for burn patients with thumb loss. It is comprised of a thumb post attached to a thumb base (or thumb spica). For realistic and aesthetic purposes, the thumb post was formed by using his uninvolved right thumb and carved with fingernail markings and creases. The splint was positioned for optimal opposition to his left middle finger. In preparation for home use, he was instructed in donning and doffing the splint and practiced using it for one-handed tasks and those involving bilateral integration skills. A QuickDASH reassessment was completed during his follow up visit six weeks later.

**Results:**

Patient self-reported an initial disability score of 77.3% on the QuickDASH. At his next visit, the patient reported daily use of the opposition splint at home to perform one-handed and bimanual activities such as dressing, cutting food, washing dishes, and folding laundry. He also showed decreased disability with a score of 59.1% on the QuickDASH, an 18.2% difference from the first assessment. This is greater than the Minimal Clinical Important Difference of 15.91 points, which is used to detect the smallest change in the outcome measure.

**Conclusions:**

Amputation of this patient’s thumb contributed to his high level of disability. With the low likelihood of obtaining a permanent prosthesis, an opposition splint helped increase his independence and daily participation in activities. After six weeks, the patient showed a positive change in status with a decreased disability as indicated on his reassessment. This device can potentially serve as a long-term prosthetic option.

**Applicability of Research to Practice:**

An opposition splint helped serve as a thumb replacement to improve independence in ADLs/IADLs in the presence of a funding challenge. Periodically evaluating a patient’s abilities with a new functional device is important to assess its effectiveness.

**Funding for the Study:**

N/A